# Characterization and identification of the xylanolytic enzymes from *Aspergillus fumigatus* Z5

**DOI:** 10.1186/s12866-015-0463-z

**Published:** 2015-06-23

**Authors:** Youzhi Miao, Juan Li, Zhizhuang Xiao, Qirong Shen, Ruifu Zhang

**Affiliations:** Jiangsu Key Lab and Engineering Center for Solid Organic Waste Utilization, National Engineering Research Center for Organic-based Fertilizers, Nanjing Agricultural University, Nanjing, 210095 People’s Republic of China; Key Laboratory of Microbial Resources Collection and Preservation, Ministry of Agriculture, Institute of Agricultural Resources and Regional Planning, Chinese Academy of Agricultural Sciences, Beijing, 100081 People’s Republic of China; Viland Biotech Group, Qingdao, 266101 China

**Keywords:** Xylan, Xylanase, *Aspergillus fumigatus*, Characterization, Identification

## Abstract

**Background:**

Plant biomass, the most abundant natural material on earth, represents a vast source of food and energy in nature. As the main component of plant biomass, xylan is a complex polysaccharide comprising a linear β(1,4)-linked backbone of xylosyl residues substituted by acetyl, arabinosyl, glucuronysyl and 4-*O*-methylglucuronycyl residues.

**Results:**

*Aspergillus fumigatus* Z5 is an efficient plant biomass depolymerization fungus. In this study, its crude xylanolytic enzymes were characterized and identified by two-dimensional gel electrophoresis (2-DE). The optimal temperature for the crude xylanases was close to 60 °C, the highest xylanase activity was achieved at pH ranged from 3 to 6, and the crude xylanases also showed a very broad region of pH (3–11) stability. The maximal xylanase activity of 21.45 U · ml^−1^ was observed in the fourth day of cultivation at 50 °C and 150 rpm with 2 % xylan as the sole carbon source. Zymogram analysis indicated that there were more than seven secreted proteins with xylanase activity. In the crude enzyme, two major endoxylanases, five cellulases and several associated enzymes were identified to be involved in the hydrolysis of polysaccharides. Of the total 13 xylanase genes in the Z5 genome, 11 were observed using q-PCR to be induced by xylan, one of which, An endo-1,4-β-xylanase with a low secretion level, was also expressed and characterized. The final hydrolysis products of xylan by crude enzyme mainly consisted of xylobiose.

**Conclusions:**

This study provides a comprehensive understanding of the depolymerization of xylan by Z5 and will help to design enzymatic strategies for plant biomass utilization.

**Electronic supplementary material:**

The online version of this article (doi:10.1186/s12866-015-0463-z) contains supplementary material, which is available to authorized users.

## Background

Plant cell walls contain the majority of organic carbon on Earth and represent a vast, renewable resource for sustainable providing of human food and energy needs. Generally, the structural polysaccharides cellulose, hemicellulose and pectin constitute the main components of the plant cell wall [1, 2]. Xylan, as the most common hemicellulosic polysaccharide, however, has attracted considerable attention for its wide distribution and its contribution to the recalcitrance of the plant cell wall [3–6]. In hardwood of angiosperms, xylan accounts for approximately 15 %–30 % of the total dry weight, and this percent increases to 30 %–35 % in the cell walls of land plants [7]. Xylan provides more than one-third of the sugars for plant cell-wall biofuel production in many feedstocks, such as hardwood and grass [8], which makes the deconstruction of xylan a critical process in the utilization of plant biomass.

Biochemical conversion is more attractive than thermochemical conversion in the deconstruction of xylan because of its low cost and high selectivity for the desired end products [9]. Structural xylan is a complex polysaccharide with an average degree of polymerization between 70 and 200 in nature [7]. It comprises a linear β(1,4)-linked backbone of xylosyl residues substituted by acetyl, arabinosyl, glucuronysyl and 4-*O*-methylglucuronycyl residues, depending on the plant species and cell type [10]. Because the building blocks of xylan represent a substantial source of nutrition for life on Earth, many enzymatic strategies have evolved to hydrolyze this recalcitrant polysaccharide to sugars. The enzymes responsible for the turnover of xylan include endo-1,4-β-xylanases, β-xylosidases, α-L-arabinofuranosidases, α-glucuronidases, acetyl xylan esterases and phenolic acid esterases [11–13].

Many fungal and bacterial species have been reported to synthesize and secrete xylanases into the external environment. *Aspergillus* genus, a group of filamentous fungi, has gained increasing attention for their highly efficient secretion of xylan-degrading enzymes. Under solid state fermentation (SSF) with *Jatropha curcas* seedcake as a substrate, a xylanase activity of 6087 U g^−1^ substrate was reported in *Aspergillus niger* by Ncube *et al.* [14]. All types of commercial xylanases produced by *A. niger* are supported by different distributors, such as Sigma, Alltech and Danisco Ingredients. Xylanase structure and function, extremophilic xylanases and synergy between xylanases have been reported in *A. oryzae*, *A. nidulans* and *A. kawachii* [15–19]. *Aspergillus fumigatus* is as versatile as other model fungi in nature; however, there are few detailed studies on its degradation of plant biomass. Ximenes *et al.* [20] isolated an *Aspergillus fumigatus* strain from a hot water fountain and investigated the cellulases of this fungus. The main cost in the depolymerization of xylan to sugars is the production of xylanases and the enzymatic hydrolysis of xylan. Therefore, the screening and isolation of powerful xylanases from microorganisms is an important focus in bioenergy research.

*Aspergillus fumigatus* Z5 was isolated from plant straw compost heaps with high and thermostable lignocellulosic enzyme activities [21]. In this study, the Z5 secreted xylanases were investigated and characterized.

## Methods

### Growth conditions

*Aspergillus fumigatus* Z5 (CGMCC accession no. 3309, China General Microbiology Culture Collection Center) was stocked in 15 % glycerol cultures at −80 °C with its conidia. It was grown on potato dextrose agar (PDA) medium for 3–4 days at 37 °C for conidia production. The conidia were harvested by washing the triangular flask with 20 ml sterile ddH_2_O. After filtration to remove the mycelia, the conidia were re-suspended, and the concentration was adjusted to 1 × 10^7^ conidia · ml^−1^. A 1 ml volume of fresh conidia suspension was inoculated into 200 ml Mandels’ salt solution supplemented with 2 % (w/v) oat spelts xylan (Sigma, USA) in a 1 L flask, and the flasks were incubated at 50 °C and 150 rpm. At each sampling time, 10 ml of culture medium was taken from the 1 L flask and filtered through a 0.45 μm membrane (Beyotime, China) twice. The clear supernatant was used as the crude enzyme extract for the next steps, and the proteins in the clear supernatant were precipitated by 80 % ammonium sulfate and redissolved in sterile distilled water. To investigate the gene expression levels, the fresh conidia were incubated in Mandels’ salt solution with 2 % sucrose as the carbon source for 20 h at 50 °C and 150 rpm. Then, the mycelia were exhaustively washed with sterile distilled water and transformed into 250 ml flasks with 1 % oat spelts xylan as the inducer. After incubation for specified times, the mycelia were harvested by filtration through one layer of gauze, then washed thoroughly with sterile water and quickly frozen in liquid nitrogen for further RNA extraction.

*P. pastoris* X33 (Invitrogen, USA) was used to express an endo-1,4-β-xylanase (GeneBank accession no.: Y699_06333), and Escherichia coli Top10 (stored in our lab) was used for the plasmid construction. YPDS medium (1%yeast extract, 2 % peptone, 2 % glucose, and 1 M sorbitol, pH 6.0), which was prepared according to the Pichia expression system manual from Invitrogen, was used for screening. BMGY/BMMY (1 % yeast extract, 2 % peptone, 1.34 % YNB, 4 × 10^−5^ % biotin, and 1 % glycerol or 0.5 % methanol, pH 6.0) were used as the growth/induction medium.

### Plasmid construction and transformation, enzyme expression and purification

The open reading frame (ORF) of the endo-1,4-β-xylanase gene, excluding the native signal sequence (amino acids 1–19), was amplified by PCR from the xylan-induced cDNA using the following primer pair: 6333-5′ (CCGGAATTCGGCGTGATCGACGAACGC) and 6333-3′ (CTAGTCTAGAGAGAGCAGCAATGATGGCA). Primer 6333-5′ introduced an EcoRI (underlined) site, while primer 6333-3′ introduced an XbaI site (underlined). After digestion with EcoRI and XbaI, the PCR production was inserted into the vector pPICZαA (Invitrogen, USA). Proper constructions were confirmed by restriction digestion and DNA sequencing, and were designated as pPICZαA−6333.

The recombinant plasmid pPICZαA-6333 was linearized with PmeI (New England Biolabs, China), and was then introduced into *P. pastoris* X33 by electroporation (Gene Pulser Xcell™ Electroporation System #165-2660, Bio-Rad, USA). Expression transformants were screened from the YPDS plates containing Zeocin™ at a concentration of 100 μg/ml according to the Pichia expression system manual from Invitrogen. Ten transformants were randomly selected for protein induction. Briefly, transformants were cultured at 30 °C for 20 h at 250 rpm in 100 ml of BMGY medium; subsequently, the culture medium was centrifuged for 10 min at 3000 rpm and *P. pastoris* was recovered into 100 ml of BMMY induction medium with cultivation for 96 h. Every 24 h, add 100 % methanol to a final concentration of 0.5 % methanol to maintain induction. After 96 h of culturing, the supernatant was recovered by centrifugation. Protein extraction was carried out by ammonium sulfate precipitation. The right transformant was confirmed by its extracellular xylanase activity and SDS-PAGE. Considering the possibility that redundant amino acids have a negative influence on the enzyme activity, Myc and His6 were not added into the C-termini of the expressed protein. Enzyme purification was carried out using a Sephadex G-200 column (GE healthcare, USA). The solution was loaded onto 2.6 × 100 cm Sephadex G-200 column equilibrated with 10 mM citrate buffer (pH 5.0). The endo-1,4-β-xylanase fractions were pooled and analyzed by SDS-PAGE.

### Enzyme assay

The xylanase activity was measured according to Linton *et al.* [22] with some modifications. The substrate solution for the xylanase assays consisted of 1 % (w/v) oat spelt xylan in sodium acetate buffer (50 mM, pH 5.0). A 20 μl volume of crude enzyme or purified endo-1,4-β-xylanase was mixed with 980 μl of substrate solution and incubated for 10 min at 50 °C, the reaction was terminated by adding 1 ml of 3,5-dinitrosalicylic acid (DNS) to the mixture, and the reducing sugar released from the enzymatic reaction was estimated by the DNS method [23] with xylose as a standard. One unit of enzyme activity was defined as the amount of enzyme required to release 1 μmol of reducing sugars from the substrate in 1 min.

### Characterization of the crude enzyme and the purified xylanase

To determine the optimal temperature of the crude enzyme and the endo-1,4-β-xylanase during the hydrolysis of oat spelt xylan, the crude enzyme or purified Y699_06333 was incubated for 10 min with 1 % (w/v) oat spelt xylan in sodium acetate buffer (50 mM, pH 5.0) at different temperatures ranging from 20 to 90 °C. The thermostability of the crude enzyme was detected by incubating the enzyme solution in 50 mM sodium acetate buffer (pH 5.0) at temperatures ranging from 20 to 90 °C. Then, the residual xylanase activity of each treatment was measured by incubating with the 1 % (w/v) oat spelt xylan solution at 50 °C and pH 5.0 for 10 min using the DNS method.

The optimal pH of the crude enzyme and the expressed xylanase during the hydrolysis of xylan was investigated by incubating the mixture of enzyme and 1 % (w/v) oat spelt xylan dissolved in an appropriate buffer at different pH values: 50 mM citrate buffer (pH 3.0-6.0), 50 mM sodium phosphate buffer (pH 6.0-8.0), 50 mM Tris–HCl buffer (pH 8.0-9.0) and 50 mM glycine-NaOH (pH 9.0-11.0) [24]. The mixture in various pH buffers was incubated at 50 °C for 10 min, and the xylanase activity was determined by the DNS method. For pH stability, the enzyme was pre-incubated without substrate in buffers of different pH values for 1 h at 50 °C, and the xylanase activity was then measured under the standard conditions.

The inhibitory effect of various metal ions, surfactants and EDTA on both the crude enzyme and purified xylanase was determined using oat spelt xylan as a substrate in reaction mixtures containing 1 ml 0.1 M sodium acetate buffer (pH 5.0), 0.5 ml 1 % (w/v) xylan solution, 3 μl enzyme and inhibitor as indicated. Determination of the kinetic parameters of the purified endo-1,4-β-xylanase was carried out under optimal conditions for 10 min at xylan concentration ranging from 2.5 to 15 mg/ml. The reaction rate versus the substrate concentration was plotted, and the data were fitted to the Michaelis-Menten equation.

### RNA extraction, cDNA synthesis and quantitative PCR analysis

The frozen mycelia for each time point were disrupted by grinding in liquid nitrogen, and the total RNA was extracted using the RNeasy Plant Mini Kit (Qiagen, Germany) according to the manufacturer’s instructions. The total RNA (approximately 2 μg) from different time points was fractionated on a 1.2 % (w/v) agarose gel, stained with ethidium bromide (EB) and visualized with UV light to determine the RNA integrity, then quantified using a NanoDrop 2000 spectrophotometer (Thermo, USA). A 1 μg mass of RNA with good quality was used to synthesize cDNA using a PrimeScript™ RT-PCR Kit (TAKARA, China) according to the manufacturer’s instructions.

All quantitative PCR runs (q-PCR) were performed in triplicate on a 7500 Fast Real-Time PCR System (Applied Biosystems) using SYBR® Premix Ex Taq™ Kit (Tli RNaseH Plus) (TAKARA, China). The amplification mixture (final volume, 20 μl) contained 10 μl 2× premix Ex Taq (Tli RNaseH Plus), 0.2 μM forward and reverse primers, 0.4 μl 50× DyeII and 2 μl cDNA (100-fold dilution). The primer sequences are given in Additional file 1. The cycling conditions comprised a 30 s initial denaturation at 95 °C, followed by 40 cycles of 95 °C for 5 s and 60 °C for 30 s. A melt curve was also performed to detect the primer’s qualities. Each experiment included an amplification-inhibited control (0.015 % (w/v) SDS in reaction mixture) and a template-free control. An actin-encoding gene (GenBank Accession No: Y699_04988) and a histone-encoding gene (GenBank Accession No: Y699_07225) were chosen together as the reference genes because of their better stability among the five detected genes under the applied conditions [25]. The expression levels of the investigated genes were analyzed according to Mach-Aigner *et al.* [26]. The log-transformed data values of the relative transcript level ratios were used for the comparative data analysis for each gene. Pearson correlations and hierarchical clustering with the average linkage clustering method were used to view the whole data set by the TIGR multiexperiment viewer software (MeV, http://www.tigr.org/software).

### Protein assay, SDS-PAGE and zymogram analysis

Protein extraction was carried out by ammonium sulfate precipitation. 100 ml of the clear supernatant was placed in a beaker kept in ice and put over a magnetic stirrer stirring at low speed, and then 51.6 g of solid ammonium sulfate were slowly added to the supernatant. After stranding for 24 h at 4 °C, the sample was centrifuged for 10 min at 10000 rpm, and the supernatant was decanted off. The protein pellets were resuspended in sterile distilled water. The protein concentration was estimated with a Micro BCA protein assay kit (Beyotime, China) using bovine serum albumin as a standard. The developed color was read at 562 nm using a Multi-Detection Microplate Reader (Spectra max M5, Molecular Devices, Sunnyvale, CA). Sodium dodecyl sulfate-polyacrylamide gel electrophoresis (SDS-PAGE) was performed using a 10 % (w/v) polyacrylamide gel according to the description in Laemmli’s research [27]. PageRuler Prestained Protein Ladder (Fermentas, China) was used as a molecular weight marker. The proteins on the gel were visualized by staining with silver or Coomassie Brilliant Blue R-250 according to the manufacturer’s instructions. The zymogram analysis of xylanase was performed by the method of Jung *et al.* [28] with some modifications. Protein samples were denatured by heating at 70 °C for 10 min in sample buffer containing β-mercaptoethanol and then subjected to a normal 10 % SDS-PAGE gel containing 0.5 % (w/v) xylan. After electrophoresis, the gel was washed twice with 25 % (*v/v*) isopropanol in 50 mM sodium citrate buffer (pH 5.4, SCB) for 25 min each at room temperature, followed by two washes of 30 min each in SCB (pH 5.4). The gel was incubated in SCB (pH 5.4) at 40 °C for 10 min for reaction, and then stained with 0.5 % (w/v) Congo red containing 5 % (*v/v*) ethanol for 15 min, destained with 1 M NaCl at last. The xylanase activity was visible as a clear band against a red background.

### 2-D PAGE and mass spectrometry (MS)

The crude enzyme was centrifuged at 14000 rpm for 10 min. The supernatant was subsequently cleaned with a 2-D Clean Up kit (GE Healthcare, USA) according to the manufacturer’s protocol and resuspended directly in the rehydration solution (8 M urea, 2 % CHAPS, 2 % IPG buffer, 0.002 % bromophenol blue, 15 mM DTT). An Ettan IPGphor 3 Isoelectric Focusing System was used for the isoelectric focusing protocol. The protein sample was applied on pre-cast IPG strips (13 cm; pH 3–10 and pH 4–7) by overnight rehydration, and the first dimensional separation was performed for 38 kV-h. After focusing, the IPG strips were incubated with equilibration buffer (6 M Urea, 75 mM Tris–HCl (pH 8.8), 30 % glycerol, 2 % SDS, 0.002 % bromophenol blue and 0.5 % DTT) for 15 min and treated again with equilibration buffer including 4.5 % iodoacetamide instead of DTT. The strips were rinsed using the SDS electrophoresis buffer (25 mM Tris, 192 mM glycine, 0.1 % SDS), placed on the gels and overlaid with 0.5 % (w/v) agarose in the electrophoresis buffer. The second dimension was run on a 10 % polyacrylamide gel at a constant voltage of 80 V for 30 min followed by a constant voltage of 120 V for 4 h at 14 °C using SE 600 Ruby (GE Healthcare, USA). After finishing, the gels were stained with silver according to the protocol from GE Healthcare.

Mass spectrometry analysis was performed according to Yan *et al.* [29]. Briefly, the protein spots of interest were excised from the preparative gels, washed three times with ddH_2_O, and then destained twice with 100 mM NH_4_HCO_3_ in 50 % acetonitrile followed by the addition of 50 % acetonitrile. After drying down with a vacuum centrifuge, the gel was reduced with 10 mM DTT in 100 mM NH_4_HCO_3_, alkylated with 40 mM iodoacetamide in 100 mM NH_4_HCO_3_, and then dehydrated with 100 % acetonitrile and dried in a vacuum centrifuge twice. The gel was digested overnight at 37 °C with sequencing grade modified trypsin (Promega, Madison, USA) in 50 mM NH_4_HCO_3_. The peptides were extracted three times using 0.5 % TFA in 50 % acetonitrile. The extracts were combined and lyophilized. The resulting lyophilized tryptic peptides were dissolved in 5 mg/ml α-cyano-4-hydroxycinnamic acid in 0.1 % TFA and 50 % acetonitrile. The peptides were then analyzed on a MALDI-TOF/TOF mass spectrometer 4700 Proteomics Analyzer (Applied Biosystems, CA, USA). The data were analyzed using GPS Explorer software (Applied Biosystems). The National Center for Biotechnology Information nonredundant (NCBInr) and fungi were selected as the database and taxonomy, respectively. Only significant hits, as defined by probability analysis (p < 0.05) in the MASCOT program included in GPS Explorer software, were accepted. The protein family was classified by InterproScan analysis (http://www.ebi.ac.uk/interpro/). The existence of signal peptide sequences was determined using SignalP 4.0 (http://www.cbs.dtu.dk/services/SignalP/).

### Thin-layer chromatographic analysis of xylanases

To investigate the final products released from the hydrolyzed xylan and several oligosaccharides, the hydrolysis products obtained from the substrate were analyzed by thin-layer chromatography (TLC) as described by Liu *et al.* Briefly, 10 μg of crude enzyme from different cultivation-time points was added to 100 μl 1 % (w/v) xylan dissolved in 50 mM sodium acetate buffer (pH 5.0), and the reaction was carried out at 50 °C for 20 h for complete hydrolysis. A 20 μl volume of the supernatant was loaded onto a silica gel 60 F254 (0.22 mm, Merck, Germany) and separated by a solvent system consisting of n-butanol-acetic acid-water (3:2:2, *v/v*). The plate was subsequently visualized by spraying with a mixture of methanol and sulfuric acid (9:1, *v/v*) and heated at 85 °C for 8 min. For the expressed endo-1,4-β-xylanase (Y699_06333), 5 μg of purified enzyme was added to 10 μl of various substrates containing 1 mg/ml of xylobiose (xy2), xylotriose (xy3), xylotetraose (xy4), xylopentaose (xy5), or xylan, and the reaction was carried out at 50 °C for 2 h.

## Results

### Time course of xylanase production

**Fig. 1:**
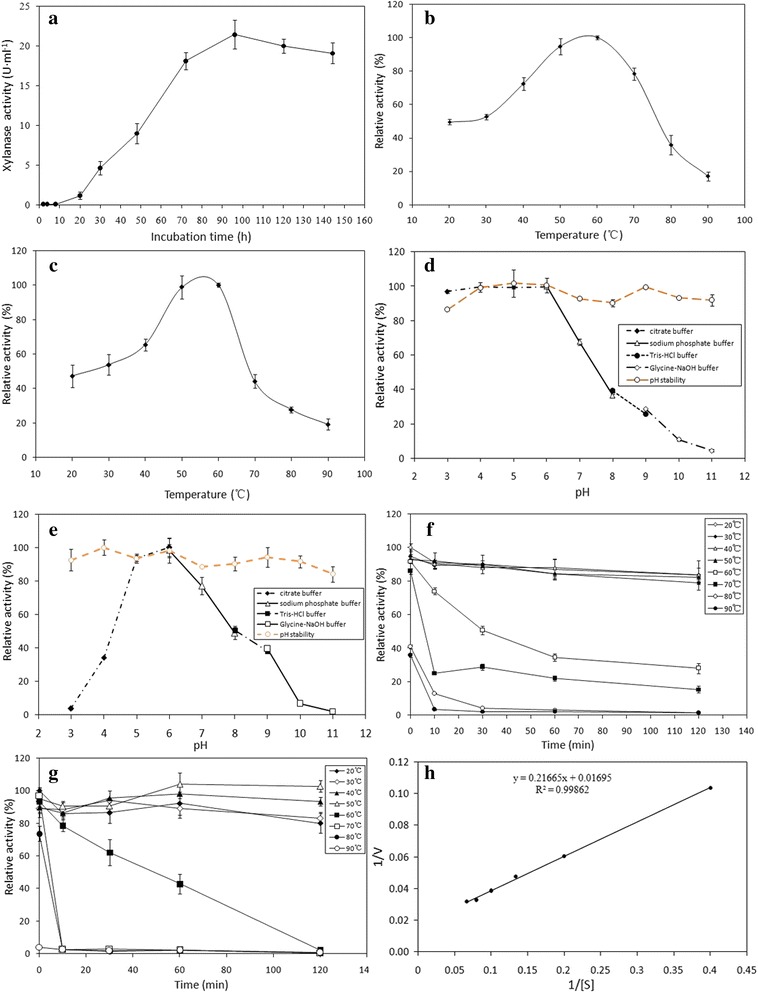
Properties of xylanases from *A. fumigatus* Z5. (**a**) Time course profile of xylanase production by *A. fumigatus* Z5 with the oat spelt xylan as the substrate. (**b**) Effect of temperature on xylanase activity of the crude enzyme. (**c**) Effect of temperature on xylanase activity of the purified endo-1,4-β-xylanase. (**d**) Effect of pH on the xylanase activity and pH stability of the crude enzyme. The enzyme was incubated at 50 °C for 10 min, with 1 % oat spelt xylan dissolved in citrate buffer (pH3.0, 4.0, 5.0 and 6.0), sodium phosphate buffer (pH6.0, 7.0 and 8.0), Tris–HCl buffer (pH8.0 and 9.0) or Glycine-NaOH buffer (pH9.0, 10.0 and 11.0). The brown line indicates the pH stability of the crude enzyme; the crude enzyme was pre-incubated without substrate in buffers of different pH for 1 h at 50 °C, and then the xylanase activity was measured under the standard conditions. (**e**) Effect of pH on the xylanase activity and pH stability of the purified endo-1,4-β-xylanase. (**f**) The thermal stability of the xylanase in the crude enzyme. The enzyme was incubated at pH 5.0 and at 20 °C (◇), 30 °C(◆), 40 °C(△), 50 °C(▲), 60 °C(□), 70 °C(■), 80 °C(○), or 90 °C(●). (**g**) The thermal stability of the purified endo-1,4-β-xylanase. (**h**) Kinetic curve of oat spelt xylan hydrolyzed by expressed endo-1,4-β-xylanase. The results are the mean of five replicates, and the bars indicate the standard error of three replicates

The time courses of xylanase activity are shown in Fig. 1a with oat spelt xylan and ammonium sulfate as the sole carbon and nitrogen sources, respectively. The xylanase released into the medium increased quickly during the first 4 days. A maximal xylanase activity of 21.45 ± 1.379 U · ml^−1^ was observed on the fourth day when incubated at 50 °C and 150 rpm; after that, the xylanase activity decreased.

### Effect of pH and temperature on the activity and stability of the crude enzyme

The effect of temperature on the xylanase activity of crude enzyme is shown in Fig. 1b. The optimal reaction temperature of the crude enzyme for xylanase was close to 60 °C. The thermal stability results indicated that after 2 h of incubation at pH 5.0, the crude enzyme maintained more than 80 % of the original xylanase activity at temperatures ranging from 20 to 50 °C, but at 80 and 90 °C, the xylanase lost nearly all activity after incubation for only 10 min (Fig. 1f).

The effect of pH on the xylanase activity of the extracellular crude enzyme was investigated in the pH ranged from 3.0 to 11.0, as shown in Fig. 1d. The optimal pH for xylanase activity ranged from pH 3.0 to pH 6.0, which indicated that high concentration of hydrogen ions had a weak effect on the xylanase activity of the crude enzyme. The pH stability assays showed no significant loss of xylanase activity in the crude enzyme during 1 h of pretreatment in the pH ranged from 3.0 to 11.0.

### SDS-PAGE and zymogram analysis of the xylan-induced crude enzyme

**Fig. 2:**
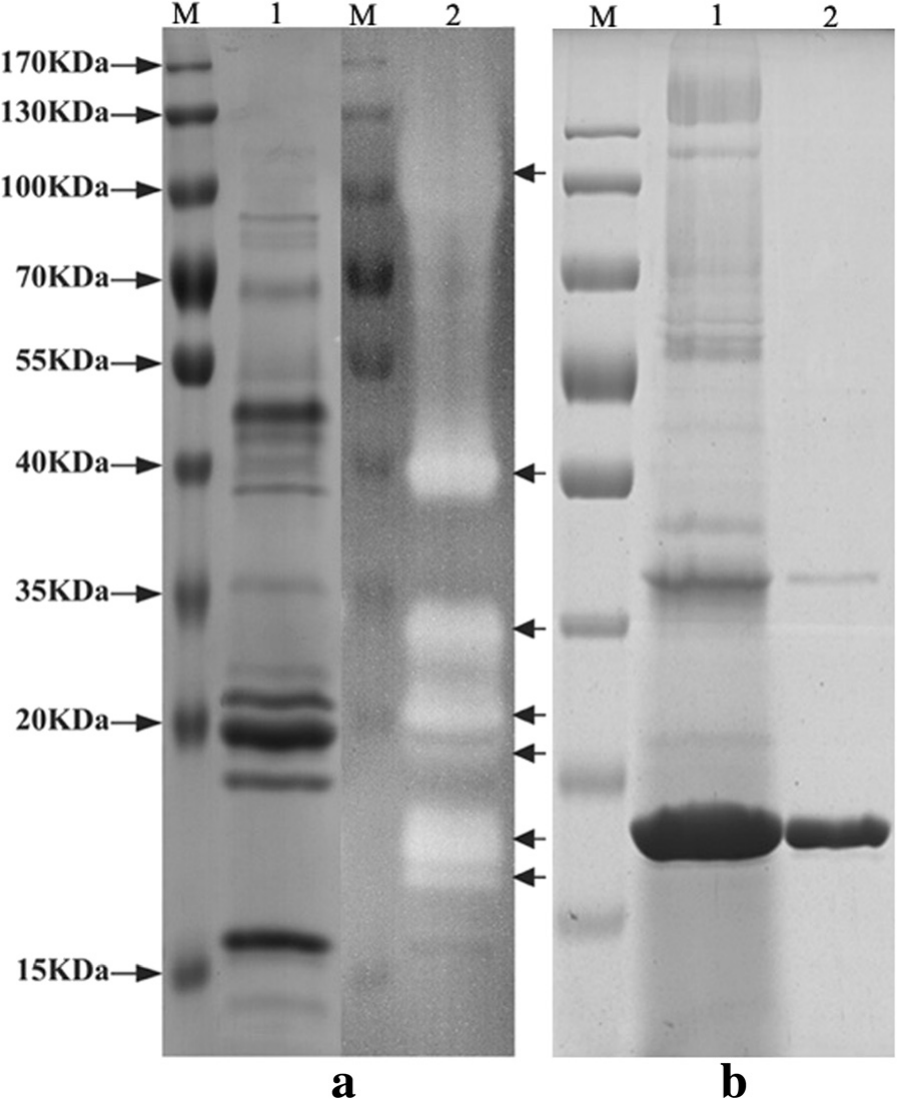
SDS-PAGE and zymogram analysis of the crude enzyme and the purified endo-1,4-β-xylanase. a-M, protein molecular weight makers; **a**-lane 1, crude enzyme stained with Coomassie Brilliant Blue R-250; a-lane 2, zymogram analysis of the xylanase. **b**-M, protein molecular weight markers; b-lane 1, culture supernatant of the endo-1,4-β-xylanase; b-lane 2, purified endo-1,4-β-xylanase

The components of the secreted proteins induced by oat spelt xylan were detected by SDS-PAGE. Fifteen visible bands were detected after staining by R-250 in the gel (Fig. 2a). The protein bands were examined for their ability to hydrolyze the xylan incorporated into the gel. Six distinct bands and a smear in the region of high molecular weight showing xylanase activity were detected in the gel. The smear region remained stable when the amount of protein sample and/or the duration of the reaction decreased. The zymogram analysis indicated that more than seven types of proteins showing xylanase activity were secreted into the medium by *A. fumigatus* Z5 under these conditions.

### 2-DE analysis and mass spectrometry of the extracellular protein induced by xylan

**Fig. 3:**
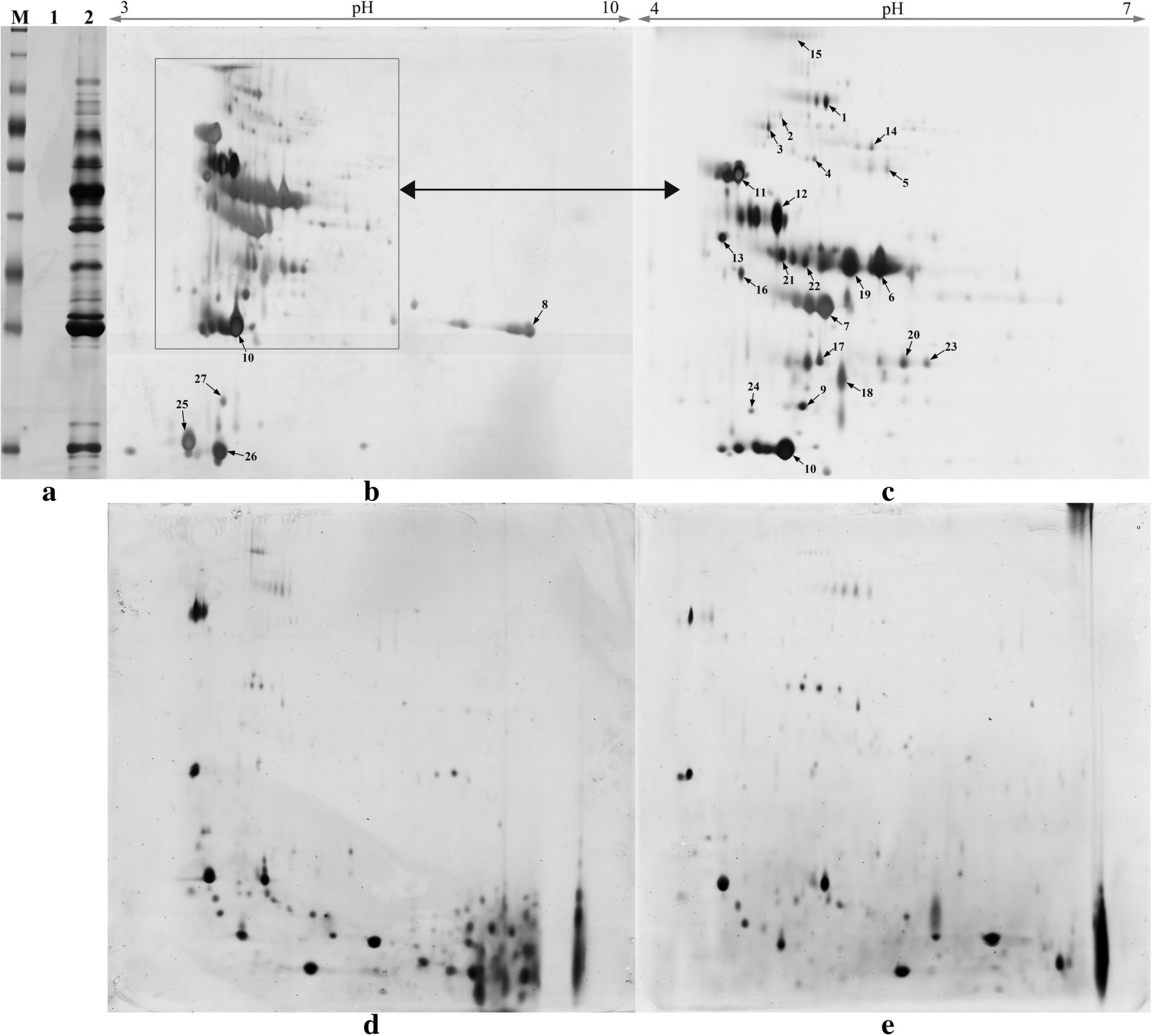
2-DE analysis between xylan-induced crude enzyme and the control induced by sucrose. (**a**) SDS-PAGE of crude enzyme. a-M, protein molecular weight markers; a-lane 1, blank sample, a-lane 2, crude enzyme stained with silver. (**b**) 80 μg of crude enzyme was loaded onto a pH 3–10 IPG strip (13 cm, linear). SDS-PAGE was performed using a 10 % gel. (**c**) Another 2-DE was performed with a pH 4–7 IPG strip (13 cm, linear) to separate the proteins with acidic isoelectric points, as shown by the region in the rectangle in b. A total of 27 identified protein spots were marked out with black arrows (**d**) The control (glucose-induced crude enzyme) was separated by a pH 3–10 IPG strip (13 cm, non-linear) (**e**) the control was separated by a pH 4–7 IPG strip (13 cm, non-linear)

To identify the secreted xylanase components from strain Z5, two-dimensional gel electrophoresis (2-DE) and MALDI-TOF-MS/MS were used for protein separation and identification, respectively. The 2-DE separation was first carried out using 13 cm immobilized pH gradient dry strips (IPG) with a linear pH 3–10 gradient. The results showed that most of the extracellular proteins were located in the pHs ranged from 4 to 6 and molecular weights ranged from 20 KDa to 70KDa, and only several points were found to have alkaline isoelectric points (Fig. 3b). Thus, another IPG strip (13 cm, pH 4–7, Fig. 3c) was used to efficiently separate those proteins having low isoelectric points. As the control, D-glucose (2 %, w/v) was used as the sole carbon source for the cultivation of Z5’s conidia. 2-D separation results of the extracellular proteins in the fourth day’s cultural solution were showed in Fig. 3d (pH 3–10) and Fig. 3e (pH 4–7). On the contrary to xylan-induced results, there were only several protein spots within the molecular weights range from 20 KDa to 70 KDa in control. Instead, most secreted proteins in control had a property of low molecular weight.

In total, 27 protein spots (Fig. 3b&c) were selectively excised from the 2-DE gel (xylan-induced) and identified by MALDI-TOF-MS/MS. The detailed results are shown in Additional file 2. There were 11 spots with the high secretion levels in the 2-DE gel, of which 6 were involved in xylan degradation, including one endo-1,4-β-xylanase (GeneBank accession no.: Y699_04481) matched to spots 6 and 19, and another endo-1,4-β-xylanase (Y699_06333) matched to spot 8. Several types of associated xylanolytic enzymes (spots 9, 14, 15, 21 and 22) were also secreted by *A. fumigatus* Z5. In the region of high molecular weight range from 70 kDa to 130 kDa, where the smear of xylanase activity appeared in the zymogram experiment (Fig. 2a), there was only one α, α-trehalose glucohydrolase that was potentially responsible for the xylanase activity. Three oxidases (Arb1, Arb2 and MreA matched to spot 2, spot 4 and spot 5, respectively) and one cellobiose dehydrogenase (matched to spot 1) were also identified in this region. The identification results also indicated that xylan could induce some cellulase genes, including three endoglucanases and two cellobiohydrolases, among which two endoglucanases (GeneBank accession no.:Y699_06174 matched to spot 10 and Y699_02044 matched to spot 12) were present at relatively high secretion levels in the medium.

### Dynamic expressions of xylanases in *A. fumigatus* Z5

**Fig. 4:**
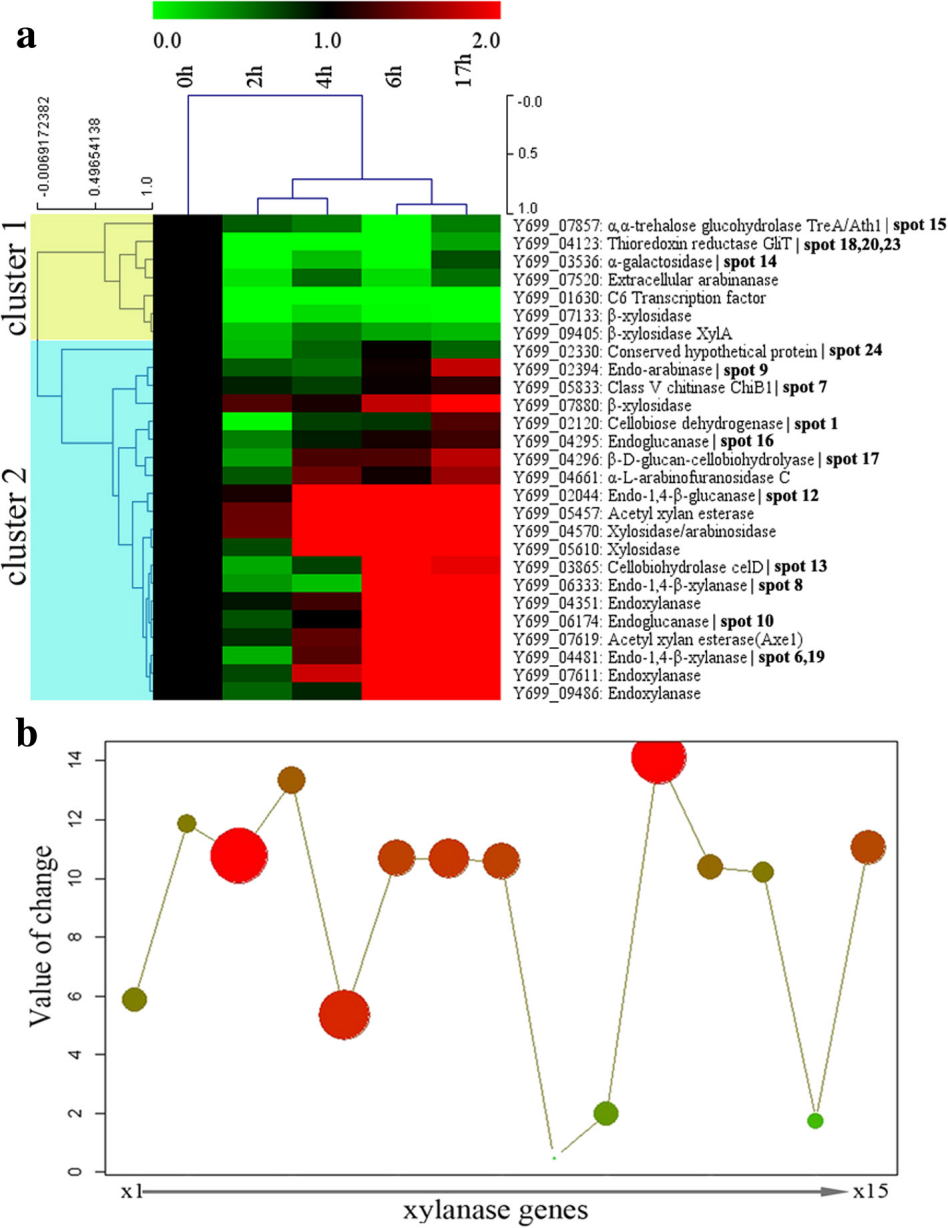
Clustering analysis of xylanase genes induced by oat spelt xylan in *A. fumigatus* Z5. (**a**) All xylanases and the some protein-identified genes were investigated under the induction of xylan at time points of 0 h, 2 h, 4 h, 6 h and 17 h after sucrose was replaced with xylan. By hierarchical clustering, two clusters were obtained; cluster 1 had no obvious changes in expression levels, but the genes in cluster2 were significantly increased. (**b**) The role of each xylanase gene in the degradation of xylan was determined by both the rate of increase under induction and its expression level. The solid circles indicate genes; the more abundant genes are colored in red and the less ones are colored in green. The diameter of the circle indicates the gene’s expression level, the y-axis indicates the rate of increase, and the x-axis represents the gene IDs for each solid circle, for which the corresponding enzyme can be found in Additional file 3

As an efficient plant biomass degradation strain, the *A. fumigatus* Z5 genome was sequenced and annotated (unpublished data, NCBI accession number: AZZA00000000). All the annotated xylanase genes in the genome and several xylan-induced secreted protein genes identified in the 2-DE gel analysis (Fig. 4a and Additional file 3) were investigated for their expression dynamics under the induction of xylan. The expression pattern formed two distinct clusters, cluster 1 and cluster 2. Cluster 1 included genes that underwent no obvious changes in their expression level. In cluster 2, expression levels of nineteen genes increased rapidly with time, including five endoxylanase genes and three xylosidase genes. Two acetyl xylan esterase genes and one α-L-arabinofuranosidase gene were also detected to be functional in this condition. The q-PCR results also confirmed that the identified cellulose-degraded enzymes in the 2-DE gel really could be induced by xylan.

Combining the expression level of each gene and its rate of change when induced by xylan, Fig. 4b was drawn to show the participation levels of those genes involved in xylan degradation by *A. fumigatus* Z5. Four endoxylanases (GeneBank accession no.: Y699_04481, Y699_06333, Y699_07611 and Y699_09486), two xylosidases (GeneBank accession no.: Y699_04570 and Y699_05610), one acetyl xylan esterase (GeneBank accession no.: Y699_05457) and one α-L-arabinofuranosidase (GeneBank accession no.: Y699_04661) were found to be the important participators in xylan degradation by strain Z5, and this will be helpful for the design of enzyme cocktail used for biofuel production.

### Hydrolyzed production of xylan by the xylan-induced crude enzyme of *A. fumigatus* Z5

**Fig. 5:**
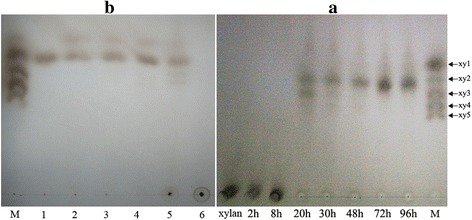
TLC analysis of the hydrolyzed products. (**a**) A 1 % xylan solution was cultivated with the crude enzyme from different incubation times, respectively, for 20 h of complete hydrolysis at pH5.0 and 50 °C. a, xylan; 2 h, 8 h, 20 h,30 h,48 h,72 h and 96 h stand for the products hydrolyzed by the crude enzyme in that time; M, mixtures of five different oligosaccharides (xy1 xylose, xy2 xylobiose, xy3 xylotriose, xy4 xylotetraose, xyl5 xylopentaose); The bottom spots in the lanes of a, 2 h and 8 h represent xylan polymer. (**b**) M, mixtures of five different oligosaccharides. 1, xylobiose treated by enzyme; 2, xylotriose treated by enzyme; 3, xylotetraose treated by enzyme; 4, xylopentaose treated by enzyme; 5, xylan treated by enzyme; 6, xylan

The products from oat spelt xylan treated with the crude enzyme over different time courses were analyzed by TLC, and the results are shown in Fig. 5a. The results indicated that in the early growth stage of Z5, there were no any proteins involved in xylan degradation secreted into the culture medium. After 20 h of cultivation, the secreted enzymes could hydrolyze xylan into the final products, xylotriose and xylobiose. As the time went on, higher xylanase activities were showed to produce more products. The final products of xylan hydrolysis by the crude enzyme secreted strain Z5 were confirmed to be xylobiose and xylose, and the former had a much higher concentration in the medium, which indicated that there was no any xylosidase secreted. An interesting result is that the final degraded products by the crude enzyme of 72 h and 96 h seemed to be located in the middle of xylobiose and xylotriose size, which indicated that oat spelt xylan has a complex structure.

### Expression of the endo-1,4-β-xylanase gene and the properties of its product

The identified endo-1,4-β-xylanase (Y699_06333, spot 8), having the alkaline isoelectric point and low secretion level, was chosen to be expressed in *P. pastoris* X33. The gene expression vector (pPICZαA-6333) was constructed as described in the part of materials and methods. The correct *P. pastoris* transformants were screened, cultured and induced with methanol for 96 h, and the supernatants were collected to determine xylanase activity. As no 6 × His-tag on the C-terminus of the expressed enzyme, the protein was purified by a Sephadex G-200 column. The endo-1,4-β-xylanase was purified 0.28-fold with 82.5 % yield.

SDS-PAGE showed that a protein, approximately 30 KDa, was secreted into the culture medium by pPICZαA-6333 transformant (Fig. 2b). The maximal xylanase activity toward oat spelt xylan occurred at pH 6.0 for the expressed endo-1,4-β-xylanase (Fig. 1e), indicating that this protein was acidic xylanase. The effect of temperature on the xylanase activity is shown in Fig. 1c, and the optimal temperature of purified endo-1,4-β-xylanase for xylanase activity was 55 °C. the pH stability of the purified enzyme is shown in Fig. 1e, indicating that it was stable in various solutions with pH ranging from 3.0 to 11.0. the thermal stability result (Fig. 1g) indicated that the enzyme maintained more than 80 % of the original activity at temperatures ranged from 20 °C to 50 °C, but at 70 °C, 80 °C and 90 °C, xylanase activity loosed rapidly. Compared to enzyme mixture in the crude enzyme, this purified xylanase showed a narrower temperature curve (between Fig. 1b and Fig. 1c), weaker temperature stability (between Fig. 1f and Fig. 1g) and smaller range of the optimal pH (between Fig. 1d and Fig. 1e).

The relative activities of the enzymes after incubation with various metal ions and chemical reagents are shown in Table 1. The results revealed that the activity of the purified enzyme was strongly inhibited by Fe^3+^, Mn^2+^, Cu^2+^, SDS and EDTA, whereas Li^+^, Ba^2+^, Ca^2+^ and Tween 80 enhanced the activity of the enzyme significantly. The crude enzyme showed a stronger resistance to those metal ions which strongly inhibited the activity of purified xylanase.

**Table 1:** Effect of various metal ions, chemical agents and chelating agent on the activity of purified xylanase and the crude enzyme. Results are the mean of three replicates

Effectors	Relative activity (%)	
	Endo-1,4-β-xylanase	Crude enzyme
Control	100	100
Metal ions (1 mM)		
Li^+^	108.9 ± 1.3^a^	88.5 ± 3.1
Fe^3+^	74.9 ± 2.4	91.8 ± 1.2
Fe^2+^	102.5 ± 0.8	102.6 ± 0.9
Ba^2+^	107.6 ± 2.6	108.1 ± 2.5
Ni^2+^	103.9 ± 1.2	89.5 ± 1.1
Mn^2+^	86.8 ± 3.2	91.5 ± 3.4
Mg^2+^	98.1 ± 1.7	95.7 ± 1.3
Ca^2+^	105.7 ± 2.8	100.0 ± 0.8
Al^3+^	93.5 ± 0.2	95.1 ± 2.1
Zn^2+^	92.2 ± 1.6	96.9 ± 1.7
Co^2+^	101.0 ± 1.9	97.6 ± 2.4
Cu^2+^	45.5 ± 2.7	69.0 ± 3.8
Surfactants SDS (0.1 %, w/v)	12.8 ± 2.5	66.5 ± 3.2
Triton X-100 (0.05 %, w/v)	101.7 ± 0.5	101.0 ± 1.9
Tween 80 (0.05 %, w/v)	107.6 ± 1.9	101.5 ± 1.7
Clelating agent EDTA (10 mM)	50.6 ± 3.1	80.5 ± 2.4

^a^ Standard deviations of relative activity

The specific activity of purified Y699_06333 towards oat spelts xylan was 26.52 ± 0.07 U/mg. The *K*_*m*_ and *V*_*max*_ values, using oat spelts xylan as the substrate, were 12.78 mg/ml and 59.00 μmol/min/mg, respectively (Fig. 1h). Products of different substrates treated by the expressed xylanase were analyzed by TLC. The results are shown in Fig. 5a and indicate that the endo-1,4-β-xylanase can hydrolyze all xylo-oligosaccharides except xylobiose. Hydrolyzed products of the endo-1,4-β-xylanase contained xylose, xylobiose, xylotriose and xylotetraose when xylan was used as the substrate.

## Discussion

Lignocellulosic biomass consists of cellulose (30-50 % dry weight), hemicellulose (20-40 %), lignin (15-25 %), ash and other components (3-10 %) [30]. While only cellulose and hemicellulose can be hydrolyzed to individual sugar molecules and then fermented to biofuel. This made the hydrolysis of polysaccharides by the cellulases and hemicellulases to be the key step in lignocellulosic biomass utilization. In the other hand, lignocellulosic biomass is structurally composed of cellulose fibres reinforced by a matrix of hemicelluloses (xylan, heteroxylans and glucomannans) impregnated with lignin, of which the chemical composition and structural organization contributes to the recalcitrance of plant biomass [31]. So cellulases and xylanases can be corporate together in the depolymerization of this complex natural material by synergetic work. In this study, *A. fumigatus* Z5 was an efficient plant biomass degrader. A xylanase activity of 21.45 U · ml^−1^ was observed under our research conditions. The crude xylanases had an optimal reaction temperature of nearly 60 °C, and were more stable than many other strains reported previously in terms of their pH and thermostability. In the process of xylan utilization, five endoxylanase and three β-xylosidase genes were induced by xylan, and two of the five endoxylanases were detected and identified in the 2-DE gel, which were proposed to be involved in the depolymerization of xylan into the final products of xylobiose and xylose. Xylobiose would be transported into the cell by special sugar transporters [32] and hydrolyzed into xylose by these two induced intracellular β-xylosidases [33] for further metabolism. The results provided a comprehensive understanding of xylan degradation by *A. fumigatus* Z5 and will help to design enzymatic strategies for plant biomass utilization.

The 2-DE separation experiment revealed that the isoelectric points for nearly all xylan-induced extracellular proteins were located in the pH range from 4 to 6. Xylanase activities in the crude enzyme were consistently very stable from pH 3.0 to 6.0. Meanwhile, Liu *et al.* reported previously that the cellulases secreted by *A. fumigatus* Z5 had an optimal pH of 5.0 and were also stable at pH 4.0-6.0 [34]. These results indicated that the lignocellulosic enzymes secreted by Z5 usually have an optimal pH close to their isoelectric point, which can be attributed to the adsorption between the enzyme and lignocellulose as well as to the electrostatic repulsion between the bound proteins [35]. Xylan and cellulose are polymerized by xylose and glucose residues, respectively, which usually have no charge. The low charge of enzymes would facilitate their adsorption onto polysaccharide backbones and reduce the electrostatic repulsion between the enzymes on the surface of the structural polysaccharides, and this could enhance the enzymatic activities. This phenomenon should be considered in the design of enzymatic strategies for plant biomass utilization.

For the full depolymerization of xylan to release sugars, at least two enzymes are required: endo-1,4-β-xylanase and β-xylosidase. The former cleaves the glycosidic bonds in the xylan backbone, reducing the degree of polymerization of the substrate. Initially, the main hydrolysis products are β-D-xylopyranosyl oligomers, and then small molecules, such as mono-, di- and trisaccharides of β-D-xylopyranosyl, may be produced [36]. The best substrate for β-xylosidase is xylobiose, and its affinity for xylooligosaccharides is inversely proportional to the degree of polymerization. Two endoxylanases (Y699_04481 and Y699_06333) were identified in the crude enzyme in this study, and Y699_04481was found in 2-DE gel analysis to be the most abundant xylan-degrading enzyme. They both belong to the glycoside hydrolase family 10, but Y699_04481 has a carbohydrate-binding module (CBM), which gives it high affinity to the backbone of xylan [37]. After the hydrolysis of xylan by these two endoxylanases, xylobiose appeared in the culture medium. It appeared that xylobiose would not be hydrolyzed in the extracellular medium because there are no any β-xylosidases identified in the 2-DE gel. Consistent with this analysis, the major hydrolysis product of xylan by the crude enzyme was also xylobiose. The q-PCR results showed that the expression levels of β-xylosidases Y699_04570 and Y699_05610 were highly induced by xylan, which indicated that they were two main intracellular enzymes toward xylobiose. Along with the expected xylanases found in the 2-DE gel, we also identified five additional cellulases. Phillips *et al.* [38] reported that cellobiose dehydrogenase could potentiate cellulose degradation by *Neurospora crassa*, and a homologue of this enzyme (GeneBank accession no.: Y699_02120) was also detected and identified in the crude enzyme induced by xylan.

The zymogram analysis of the crude enzyme showed abundant protein bands for xylanase activity. However, only two types of endoxylanases were detected. The most interesting was a smear of xylanase activity in the high molecular weight region. The protein concentration in the smear was so low that it was necessary to increase the loading quantity of the crude enzyme in 2-DE separation to obtain enough proteins for identification. Seven sampling protein spots (spots1-5, spot 14 and spot 15) were focused in this smear region in the 2-DE gel. Unfortunately, there were no xylanases but rather a cellobiose dehydrogenase, an α,α-trehalose glucohydrolase and three oxidases that most likely contributed to the xylanase activity of the smear. However, there were still more than fifteen visible protein spots that were not detected, in which the three remaining induced endoxylanases may be found.

## Conclusions

In summary, the evaluation of xylanase activity and the zymogram analysis results indicated that *A. fumigatus* Z5 has great potential for use in xylan deconstruction. The combination of q-PCR, 2-DE protein identification and TLC is an effective technique for understanding the mechanism by which *A. fumigatus* Z5 secretes xylanases and utilizes structural xylan. The cultivation of *A. fumigatus* Z5 with xylan indicated that the crude enzyme actually was a complex mixture consisting of xylanases, cellulases, chitinases, glycoside hydrolases, oxidoreductases, hypothetic proteins and other unidentified proteins.

## Additional files

Additional file 1:
**Primers used for qPCR in this study.**
Additional file 2:
**Identified extracellular proteins induced by xylan.** For each identified protein, the matched peptide sequences and scores were listed. Additional file 3:
**Expression dynamics of xylanase genes and 2-DE identified protein genes in** ***A. fumigatus*** **Z5 under the xylan induction.** Gene’s relative expression levels were given out at 0 h, 2 h, 4 h, 6 h, and 17 h after xylan being sole carbon source.
