# Supplementary material for: Characterization and identification of the xylanolytic enzymes from Aspergillus fumigatus Z5
Source: BMC Microbiol. 2015 Jun 23;15:126. doi: 10.1186/s12866-015-0463-z (PMC4477485; doi:10.1186/s12866-015-0463-z)
Supplement: Additional file 1: — Primers used for qPCR in this study. [file 12866_2015_463_MOESM1_ESM.docx]

**Additional file 1.** Primers used for qPCR in this study

| Gene ID | Forward primers(5’→3’) | Reverse primers(5’→3’) |
| --- | --- | --- |
| Y699_01630 | TTTCCGAGAGCCGTACCACTATC | AACCGCAAGAGACGCAACTAAC |
| Y699_02044 | GTGTCAGTTGTGCAGTCCAGTTC | TCGTGGTCGTAGTTGTGGTCG |
| Y699_02120 | GGAACTGACGCTGAACGACAAC | GCTCACTGGCAGACACAAGATAC |
| Y699_02330 | CGCTGAAGAAGGTTGTGGAGAATC | ACCAGAACCAATCCTCCGCAG |
| Y699_02394 | CCGTATGTCAAGACTGGTCAGAAAC | GGACTCACAGAACGCATGGAAG |
| Y699_03536 | ACTACGCCAACACGGTCTTCA | GTTGCTGGTGGGACTGAGGTA |
| Y699_03865 | AGATGACCGTAGTGACCCAGTTC | GAGACAGAGTTGGCAATCACCTTA |
| Y699_04123 | TTCCGAGTCCGTCACCATCTAC | CGAATTTCAGTCCCGAGGCAC |
| Y699_04295 | GCTATCTCTCGTTGTTGAAGCCGTA | TGTGGTCGTGGTAGTGGTGG |
| Y699_04296 | TCAGCGGCAACTCCATCACC | CACAGGGACATGACGAGAACCA |
| Y699_04351 | CATCCAGGGCACGGCTACTT | CCGCATTTCTCCACGCATCAA |
| Y699_04481 | GCACCACAACGACCACTACTACTA | GTCCAGCCAATACCGCCACA |
| Y699_04570 | TGGACATTGTGGAGGCGACTT | GTAGCGTCATAGTAGATTCGGAGGA |
| Y699_04661 | ACTTCACCGCTGTCTTCTGGAT | ACTGGCTCTTTGTTCTGCTTCTTC |
| Y699_04988 | GCTCGTCGTGGTGGTGTCAA | TGGCGTGCTCAGTGTAGGTG |
| Y699_05457 | ACGACTACTACTACAACGCCTCC | CCATTGCCGCCACATTGACC |
| Y699_05610 | GCTGGACGACGCATCATTCTATT | CAATCTCCCGCATCTTCACACTC |
| Y699_05833 | ACGACCAGCAAGCGAACGA | AGAAGCCACCGTCAGAAGGAAG |
| Y699_06174 | CTGGTGTTTGGGCTGATGATGAA | AGGTTACCCGCAGAGTGAAGG |
| Y699_06333 | GAGAACCACATCACCACCGTCA | GGCAATCCGCACAAAGTCCTC |
| Y699_07133 | TTCCATCGGATCTGACACTCCATAT | GGAGCGAATGCCAACCAAGAC |
| Y699_07225 | TGCCCTTGCTCCCTCGTCTA | ACCGCTCTCGTCGTACTCCT |
| Y699_07520 | AGGGCGAAACCGATGACGAG | CCATACGGCAGATGCGACAGA |
| Y699_07611 | GCCGTTCGCTACAATGCTCC | TTCCCAGGTTCATGCCCAGTC |
| Y699_07619 | TCCTCGTCCTCCTGGCTTCA | GCTGCCTGTCCGTACTCCG |
| Y699_07623 | TCTGGTGGTTCTTCTTCTGGTGG | TTCGGACTCTATATGGCATCGGTAT |
| Y699_07857 | CCGCTACGCCACTCTGTTGA | CGTCCAGCCTTCTCTTGTTGTTG |
| Y699_07880 | GCCCTGGGACCGTATAACACA | CAAGAACAGCAACGCCACATAATC |
| Y699_09405 | CAAGACCTCCTTCAACATCCAAGAC | GCGAAGAGCATAGCAGTGTAGTC |
| Y699_09486 | GTGTTGGTGTTGTAGTCTGGGATTT | TCGGCAGCAGCATAGAAGGC |
| Y699_00150 | AGAACGACCTACAGCAACCAGT | GTATTCACACCACCAGCCACAC |
| Y699_00561 | CAAACATCCACTACGCCAAGGG | CAGGTGCCAACGACGACAATG |
| Y699_00572 | GCCTCTCCTACACGACCTTCTC | GCCAGCCACACTACCTGTATTAG |
| Y699_01298 | TGACGGAGAATGGCACGACAG | CCACCCAACATATCCCTCAAAGAAC |
| Y699_02036 | CTGAGCTACGACAATGGCAAGAC | ATTATACCAAGTCCACGCAAAGAGG |
| Y699_02067 | GACCAACGAGGCGGAACTGA | CTTCGCAAACCAAACCATCACTTC |
| Y699_02418 | AGGACCGCATTGAGTTTACCATTC | CCACCGCCAACAACCTTGAC |
| Y699_02424 | GCAAGAACAAGACCAAGAGGAACA | GGCACTTCAAATCCGCACCAG |
| Y699_02437 | CGCTTCGTCTCGCCTTATTCC | CGTTGTCAGTGTCGCCGATTC |
| Y699_02871 | ACTTCTGCGTCTGCCTCTGG | CTGGTGGTAGTAAGGGTTCTGCT |
| Y699_03689 | AGCTCGCCTTCGACCTTTGT | CCACCGCACTGACCATAGACA |
| Y699_02885 | ACCTACTACGCCGCCTACGA | GCTCAGACTCCATTCTCCCACAA |
| **Supplementary Table S1** (continued) | | |
| Y699_03907 | CAATGACAACCTGAAGGCTCTGAC | CCGAAACCCGCTCCTTACCA |
| Y699_04028 | CAGTGTTTGGGATGTGGTGGTG | AGGACAGGCTCGGTTGAGGA |
| Y699_04562 | GCTGATTTCGTCCGTGCTTGA | TTGCCTCCATACCATGCCTGAA |
| Y699_04322 | AGCCGCCACTCTTCCGATTC | CGCTGGTGTTGGAGACAGTCA |
| Y699_05110 | CCAGCAGCAGCAACCTCACT | CACCTTCTCCGTAACCAGCCA |
| Y699_05311 | TGGATGCGAGGACTGGAGTATATG | CTGCGTGTTGCTTGTGGAGG |
| Y699_05430 | CCACTCCCACCACCTTCGTTA | CCGCACTGCCCGTAAATCTTG |
| Y699_05768 | CAGTCCATCAAGCCGCACAAG | ATCTCGCACACTCTTACCCACAT |
| Y699_05825 | CTGCTAATGTGGTGAATGGCGATA | GCTGTTGGAGACGGGAGACTT |
| Y699_06723 | AGAGCGGTGTTCTAGTTCGTCAA | TACCACTTCCCAGGCGTCATTC |
| Y699_06818 | AGGTGAAGAGTTTCGCCAATGC | GAGCCAGAATAGTTCCACTTCCAAG |
| Y699_07344 | GCCGCACAACTATTCATCCTACC | GCTTTACGCTTACCATCTCCTCG |
| Y699_07518 | CTTCACCATCACCACGCAGTTC | CGGCATTGGCGATCACCTTG |
| Y699_07603 | TGGCAACGGAGGCAATGGT | CTGTAATGGCTGGCTGTCCTGA |
| Y699_07898 | AGGCTAGTGCTGTTGCTGAAGT | ACCACGAAGATACCAGCGATAGG |
| Y699_08586 | AGCGTCACCTACTGCGGAAC | GCTATGTGCCAACTCGTCTAACTC |
| Y699_08636 | ACCTGGGAGACCGCAACAAC | ACGCTGTGGATGACGACGAT |
| Y699_08637 | TTGCGTTATTCATGTCGGACTGG | AATGTTGACACTGGCGATCTGC |
| Y699_08692 | TGAGACTCCAGTGGGCATGATG | AGTGGTGGTAGTGGTGGTTGTC |
| Y699_08855 | ACACCGCCAAGCCGATAACC | CCGAATTGCCGATCACCATACC |
| Y699_09008 | ACAAGTTTCGCAAGCACCTCAC | GCATGTCATCCGATCCTCTCCA |
