# Supplementary material for: Characterization and identification of the xylanolytic enzymes from Aspergillus fumigatus Z5
Source: BMC Microbiol. 2015 Jun 23;15:126. doi: 10.1186/s12866-015-0463-z (PMC4477485; doi:10.1186/s12866-015-0463-z)
Supplement: Additional file 2: — Identified extracellular proteins induced by xylan. For each identified protein, the matched peptide sequences and scores were listed. [file 12866_2015_463_MOESM2_ESM.docx]

**Additional file 2.** Identified extracellular proteins induced by xylan

| **Spot no.** | **NCBI accession no.** | **Protein names** | **Matched peptide Sequences** | **Scores** |
| --- | --- | --- | --- | --- |
| 1 | Y699_02120 | Cellobiose dehydrogenase | RQIQWTARM\|RVVLSAGTFGSARI\|RGGPLATYLVSASERN\|RMTITANLDTVVSTLPYLRD\|RDQHDVDAVIQGLVNLQNALKG\|RIGILTQSAPNIGPIFFDEIRG | 335 |
| 2 | Y699_02126 | Conidial pigment biosynthesis oxidase Arb1 | KISVPLDHQRI\|RQIIFDTGVTQRD\|RDTIMVQNHGHAVLRF | 114 |
| 3 | Y699_01844 | Glutaminase GtaA | RQTQLHFSEVRD\|KSIFTFHVGDAVEMKV\|KGDWELFTAAVASTSTRD\|RDSVAAAGQDYLTITSLSARQ\|RDQAEWGNWYWATEHSDRM | 205 |
| 4 | Y699_02127 | Conidial pigment biosynthesis oxidase Arb2 | RYIEPQLAHSVKL\|KEASPESFLETPVYRD\|RDTFTTSPQGETWTAIRY | 104 |
| 5 | Y699_07340 | FAD/FMN-containing isoamyl alcohol oxidase MreA | KMTDEYIPRL\|KVGAGVQGIEAYRA\|RELVRPFTDGLTRL\|KSTGAGALAIWTHHLKD\|RSVVQTNNAELTAAYRA\|RLEALAPQSGAYLNEADFRQ\|RHGLAADQVLEWEVIDGQGNFLVANRD | 227 |
| 6 | Y699_04481 | Endo-1,4-β-xylanase | KNHITNVVTHYKG\|KLYYNDYNIEYSGAKA\|KGYTALGVEVAYTELDIRM\|KIDGVGLQAHFIVGSTPSQSDLTTVLKG\|RNSVFYQIIGPAYIPIAFATAAAADPDVKL | 340 |
| 7 | Y699_05833 | class V chitinase ChiB1 | KIVLGMPLYGRS\|KIVLGMPLYGRS+Oxidation(M)\|RNHNPQDLPVERL\|RSVVYFVNWAIYGRN\|KFLISYDNPQVANLKS\|KVLLSIGGWTYSPNFAPAASTDAGRK\|KVLLSIGGWTYSPNFAPAASTDAGRKN\|RTALDSYSAANAGGQHFLLTVASPAGPDKI\|RTALDSYSAANAGGQHFLLTVASPAGPDKIKV | 482 |
| 8 | Y699_06333 | Endo-1,4-β-xylanase | RIAFETARS\|RVLGEDFVRI\|KWDATEPSQGRF\|RYFGTASDQALLQKS\|RFNFAGADFLVNYAKQ\|RSGSSPLLFDSNYQPKA\|KLYINDYNLDSASYGKT\|KGQIYAWDVVNEIFNEDGSLRD\|KWLAAGIPIDGIGTQTHLGAGASSSVKG\|RYKGQIYAWDVVNEIFNEDGSLRD\|RGHTLVWHSQLPSWVSAISDKNTLTSVLKN | 578 |
| 9 | Y699_02394 | Endo-arabinase | RNDGYYVMYYSGEAKE\|RRNDGYYVMYYSGEAKE\|RVADDGFTPLGDAVPILDRD\|RLDQGGSIDPAGFLDKDGSRY | 222 |
| 10* | Y699_06174 | Endoglucanase | NTDPGIKFDIYSDLSGGYPIPGPALFNA |  |
| 11 | Y699_00170 | extracellular lipase | KLLVVSFRG\|RVTHTNDIVPRL\|KAGIPVELYGYGSPRV\|KALATFITGQGSNYRV\|RKAGIPVELYGYGSPRV\|KLTCSSGECPLVEAANTKT | 387 |
| 12 | Y699_02044 | Endo-1,4-β-glucanase | KTTLEFFKI\|RGHAVVAAGDKI\|RCGSSCETVDKTTLEFFKI\|KISIQWTAWPDSHHGPVIDYLARC | 157 |
| 13 | Y699_03865 | Cellobiohydrolase celD | RLYVQNGKV\|KHGGLSGMGKA\|KFGPIGSTYEG-\|KAFGDEDIFAKH\|KLSFVTGANVGSRL\|KKAFGDEDIFAKH\|KYGTGYCDSQCPRD\|RYAGTCDPDGCDFNPFRM\|KFINGMANVEGWEPSSSDKN+Deamidated(NQ)\|KCTAAGSCSQQSGSVVIDANWRW\|KMTVVTQFITADGTDSGALSEIKRL\|KVIANSVSNVAGVSGNSITSDFCTAQKK | 517 |
| **Supplementary Table S2** (continued) | | | | |
| 14 | Y699_03536 | α-galactosidase | RHSELYSPGKG\|RYEIMSSALARV\|RIGNDIIPAWRS\|KTFAEWGADSLKY\|KALVDLGLAELGYRY\|RYVTTDCGWSVADRL\|RIALTAGSNTITIQHKT\|KDVIGFNQDALGVSASLKR\|KDVIGFNQDALGVSASLKRR\|RDLTLDLPDVGLQYAQVARN\|KVVFGNEGGEDGFQTYAADFVGVRV\|RTPQMGWNSYNYYSCSPNEAIIRS\|RTPQMGWNSYNYYSCSPNEAIIRS+Oxidation(M)\|KYLAIDYINNEVAFSSSWGWGSNSRN\| | 638 |
| 15 | Y699_07857 | α, α-trehalose glucohydrolase | RLPVTGPLVVPNRQ\|RAANLAPLDTADSRF\|RTTFSPSAAIYPWTSGRF\|KFVGAASSDAFPNPQQTAKQ\|REQHFPIYDSVATMYSNIVQRN | 174 |
| 16 | Y699_04295 | Endoglucanase | RVQAATQWLKT\|KLNDYYSQCL-\|KTLHDAGMNIFRV\|KTLHDAGMNIFRV+Oxidation(M)\|KLPGVLGTDYIWPDASTIKT\|RYYGNIISSTDDFAAFWKT\|RASGFVWFGSNESGAEFGETKL\|KATVNAITSLGAYAVIDPHNYGRY | 329 |
| 17 | Y699_04296 | β-D-glucan-cellobiohydrolase | RFYVQNGKV\|KVVIDANWRW\|KDDSTYEMFKL\|KTFYGPGMTVDTKS\|KSLFQDQNVFEKH\|KYGTGYCDSQCPRD\|RYGGTCDPDGCDFNSFRQ\|KFTVVTQFITDDGTSSGTLKE | 391 |
| 18 | Y699_04123 | Thioredoxin reductase GliT | RDNEGHSWYGRK\|RQVETNQLFEARD\|RQLHTAVVFDSGVYRN\|KEIQQAAEESPVGASGLKF\|RGQETVGVLALGPIANPARA\|RLSESVTIYTNGNEQLAKE+Deamidated(NQ) | 267 |
| 19 | Y699_04481 | Endo-1,4-β-xylanase | KLNDWYSQCL-\|KNHITNVVTHYKG\|KLYYNDYNIEYSGAKA\|KGYTALGVEVAYTELDIRM\|KCYAWDVVNEALNEDGTFRN\|KCYAWDVVNEALNEDGTFRN+Dioxidation(W)\|KWGQCGGIGWTGPTTCVSGTTCQKL\|RNSVFYQIIGPAYIPIAFATAAAADPDVKL\|KWDATEPSQNSFSFANGDAVVNLANKNGQLMRC+2Deamidated(NQ) | 294 |
| 20 | Y699_04123 | Thioredoxin reductase GliT | KVVLATGVRD\|KFEARPIRR\|RDNEGHSWYGRK\|RDNEGHSWYGRK+Dioxidation(W)\|RQVETNQLFEARD\|RQLHTAVVFDSGVYRN\|KTQHMHNVLGWDHRN\|KEIQQAAEESPVGASGLKF\|RGQETVGVLALGPIANPARA\|RLSESVTIYTNGNEQLAKE+Deamidated(NQ)\|RQLHTAVVFDSGVYRNAKT+Deamidated(NQ)\|KTEGFLVYNPQTEVNGPFAKQ+Deamidated(NQ)\|KTQHMHNVLGWDHRNPAELRA\|KAVTPAVSMGSLAAGGLVAQLQAQALPEFRL | 496 |
| 21 | Gi\|70989361 | α-1,3-glucanasemutanase | KQQFSLLRN\|RQYASLPAQLKV\|KSANNYITEDKL\|RWQEILNLGPRF\|KLIYWYRPTPKD\|RQSAADYDDDMQRA\|RQSAADYDDDMQRA+Oxidation(M)\|RLVFCHFMIGITSNRQ\|KTFQAQAGASAFSAPMGVGKQ\|KTFQAQAGASAFSAPMGVGKQ+Oxidation(M)\|RGRPNGYESMTDEVFVVSLLKS\|KSANNYITEDKLIYWYRPTPKD\|KALGIDAFALNIGVDPYTDTQLNFAYESAARN | 429 |
| 22 | Gi\|70989361 | α-1,3-glucanasemutanase | KQQFSLLRN\|RQYASLPAQLKV\|RWQEILNLGPRF\|KLIYWYRPTPKD\|RQSAADYDDDMQRA\|KTFQAQAGASAFSAPMGVGKQ\|RGRPNGYESMTDEVFVVSLLKS+Deamidated(NQ)\|RGRPNGYESMTDEVFVVSLLKS+Deamidated\|(NQ);Oxidation(M) | 306 |
|  |  |  |  |  |
|  |  |  |  |  |
|  |  |  |  |  |
|  |  |  |  |  |
| **Supplementary Table S2** (continued) | | | | |
| 23 | Y699_04123 | Thioredoxin reductase GliT | KVVLATGVRD\|KFEARPIRR\|RKVVLATGVRD\|RDNEGHSWYGRK\|RDNEGHSWYGRK+Dioxidation(W)\|KTVIVHLGESESKT\|RQVETNQLFEARD\|RQLHTAVVFDSGVYRN\|KTQHMHNVLGWDHRN\|KTQHMHNVLGWDHRN+Oxidation(M)\|KEIQQAAEESPVGASGLKF\|RGQETVGVLALGPIANPARA\|RLSESVTIYTNGNEQLAKE+Deamidated(NQ)\|KTEGFLVYNPQTEVNGPFAKQ | 633 |
| 24 | Y699_02330 | Hypothetical protein | RWMVGGQTKW\|RWMVGGQTKW+Oxidation(M)\|RLSGTADDNTLRW\|KTVGLWASTGAEALKK\|KTVGLWASTGAEALKKV\|RITIDSSSSWNGQTMMRS\|RITIDSSSSWNGQTMMRS+Oxidation(M)\|KVVENLGANTFTDSQDWHVGELRL | 343 |
| 25 | Y699_02605 | Allergenic cerato-platanin | KWPTFGSVPGFARI\|RIGGAPTIPGWNSPNCGKC\|RVQATYEEADPSHCASGV-\|RYDNGGTSMNDVSCSNGVNGLVTKW+Oxidation(M) | 98 |
| 26 | Y699_07973 | Cell wall protein | REATVEVSLHRA\|KWVQGLFADAIKA\|KNLSVLSSELPNALEKI\|KAFDGHPAGYTTITERE\|KKPQVNDIGKSDDMLRI | 209 |
| 27 | Y699_00114 | Hypothetical protein | KYVYDLTITSAEKN\|RDLFPHWISQGGSCNTRE\|RDLFPHWISQGGSCNTRE+Dioxidation(W)\|RAFANDLTNPQLIAVTDNVNQAKG\|KGDDGPEAWKPPLTSYYCTYAKM | 150 |

*Identification of spot 10 had no protein hits in NCBInr. However, one non-assigned peptide with high sequencing score, was searched in whole protein sequences of *A. fumigatus* Z5, and successfully matched to protein Y699_06174 that is an endoglucanase.

Column Matched peptide sequences contain the peptides matched to the corresponding protein, and the peptides were separated with “|”.
