# Supplementary material for: Characterization and identification of the xylanolytic enzymes from Aspergillus fumigatus Z5
Source: BMC Microbiol. 2015 Jun 23;15:126. doi: 10.1186/s12866-015-0463-z (PMC4477485; doi:10.1186/s12866-015-0463-z)
Supplement: Additional file 3: — Expression dynamics of xylanase genes and 2-DE identified protein genes in A. fumigatus Z5 under the xylan induction. Gene’s relative expression levels were given out at 0 h, 2 h, 4 h, 6 h, and 17 h after xylan being sole carbon source. [file 12866_2015_463_MOESM3_ESM.docx]

**Additional file 3.** Expression dynamics of xylanase genes and 2-DE identified protein genes in *A. fumigatus* Z5 under the xylan induction

| **Enzyme** | **Family** | **SP** | **2-DE** | **Lg of relative transcript levels** | | | | | **GenBank** |
| --- | --- | --- | --- | --- | --- | --- | --- | --- | --- |
|  |  |  |  | **0h** | **2h** | **4h** | **6h** | **17h** | **accession no.** |
| **Xylan-degrading enzymes** |  |  |  |  |  |  |  |  |  |
| Endo-1,4-β-xylanase (x3) | GH10/CBM1 | Y | 6,19 | 0 | 0.31±0.02 | 1.33±0.00 | 3.20±0.01 | 3.24±0.02 | Y699_04481 |
| Endo-1,4-β-xylanase (x8) | GH10 | Y | 8 | 0 | 0.41±0.03 | 0.25±0.02 | 3.19±0.02 | 2.57±0.01 | Y699_06333 |
| Endoxylanase (x11) | GH11 | Y |  | 0 | 0.72±0.01 | 1.81±0.03 | 4.25±0.03 | 4.24±0.02 | Y699_07611 |
| Endoxylanase | GH11 | Y |  | NS | NS | NS | NS | NS | Y699_07623 |
| Endoxylanase (x15) | GH10 | Y |  | 0 | 0.60±0.06 | 0.86±0.10 | 2.95±0.09 | 3.34±0.11 | Y699_09486 |
| Endoxylanase (x2) | GH11 | Y |  | 0 | 0.91±0.07 | 1.25±0.10 | 3.57±0.04 | 2.99±0.08 | Y699_04351 |
| β-xylosidase XylA (x14) | GH3 | Y |  | 0 | 0.25±0.01 | 0.52±0.01 | 0.34±0.02 | 0.27±0.04 | Y699_09405 |
| β-xylosidase (x13) | GH3 | Y |  | 0 | 1.32±0.02 | 1.10±0.02 | 1.76±0.04 | 3.07±0.00 | Y699_07880 |
| β-xylosidase (x9) | GH43 | Y |  | 0 | -0.04±0.01 | 0.14±0.05 | 0.02±0.02 | -0.12±0.04 | Y699_07133 |
| Xylosidase (x7) | GH43 | N |  | 0 | 0.71±0.01 | 2.00±0.01 | 3.21±0.00 | 3.17±0.02 | Y699_05610 |
| Xylosidase/arabinosidase (x4) | GH43 | N |  | 0 | 1.42±0.02 | 2.62±0.03 | 4.02±0.01 | 3.89±0.01 | Y699_04570 |
| α-L-arabinofuranosidase C (x5) | GH62/CBM1 | Y |  | 0 | 0.66±0.01 | 1.42±0.02 | 1.06±0.01 | 1.60±0.03 | Y699_04661 |
| Acetyl xylan esterase (x12) | CE5/CBM1 | Y |  | 0 | 0.82±0.04 | 1.37±0.01 | 3.13±0.02 | 2.41±0.02 | Y699_07619 |
| Acetyl xylan esterase (x6) | CE1/CBM1 | Y |  | 0 | 1.41±0.02 | 2.01±0.02 | 2.20±0.16 | 3.22±0.11 | Y699_05457 |
| α, α-trehalose glucohydrolase | GH65 | Y | 15 | 0 | 0.64±0.03 | 0.51±0.03 | -0.18±0.03 | 0.51±0.03 | Y699_07857 |
| Extracellular arabinanase (x10) | GH43 | Y |  | 0 | 0.11±0.01 | 0.59±0.02 | 0.14±0.02 | 0.55±0.00 | Y699_07520 |
| α-galactosidase | GH27 | Y | 14 | 0 | -0.30±0.01 | 0.25±0.00 | -0.03±0.01 | 0.69±0.01 | Y699_03536 |
| Endo-arabinase (x1) | GH43 | Y | 9 | 0 | 0.64±0.03 | 0.56±0.01 | 1.07±0.01 | 1.77±0.02 | Y699_02394 |
| α-1,3-glucanase/mutanase | GH71 | Y | 21,22 | ND | ND | ND | ND | ND | Gi\|70989361 |
| **Cellulase-degrading enzymes** |  |  |  |  |  |  |  |  |  |
| Endoglucanase | GH61 | Y | 10 | 0 | 0.68±0.01 | 1.00±0.02 | 3.97±0.00 | 2.85±0.02 | Y699_06174 |
| Endoglucanase | GH5/CBM1 | Y | 16 | 0 | 0.50±0.05 | 0.89±0.01 | 1.09±0.07 | 1.24±0.03 | Y699_04295 |
| Endo-1,4-β-glucanase | GH61/CBM1 | Y | 12 | 0 | 1.10±0.06 | 2.19±0.04 | 2.14±0.06 | 2.70±0.06 | Y699_02044 |
| β-D-glucan-cellobiohydrolase | GH7/CBM1 | Y | 17 | 0 | 0.38±0.03 | 1.29±0.03 | 1.30±0.08 | 1.74±0.02 | Y699_04296 |
| Cellobiohydrolase celD | GH7 | Y | 13 | 0 | 0.34±0.04 | 0.75±0.07 | 2.38±0.02 | 1.92±0.05 | Y699_03865 |
| Cellobiose dehydrogenase |  | Y | 1 | 0 | 0.02±0.00 | 0.75±0.01 | 0.79±0.03 | 1.32±0.02 | Y699_02120 |
| **Others** |  |  |  |  |  |  |  |  |  |
| Class V chitinase ChiB1 | GH18 | Y | 7 | 0 | 0.88±0.02 | 0.75±0.00 | 1.04±0.01 | 1.18±0.04 | Y699_05833 |
| Thioredoxin reductase GliT |  | N | 18,20,23 | 0 | -1.43±0.04 | -1.49±0.01 | -1.21±0.01 | 0.36±0.02 | Y699_04123 |
| Hypothetical protein |  | Y | 24 | 0 | 0.27±0.04 | 0.61±0.05 | 1.02±0.05 | 0.61±0.03 | Y699_02330 |
| C6 Transcription factor | GH43 | N |  | 0 | -0.48±0.00 | -0.56±0.00 | -0.24±0.06 | -0.41±0.00 | Y699_01630 |
| Extracellular lipase |  | Y | 11 | ND | ND | ND | ND | ND | Y699_00170 |
| Conidial pigment biosynthesis oxidase Arb1 |  | Y | 2 | ND | ND | ND | ND | ND | Y699_02126 |
| Conidial pigment biosynthesis oxidase Arb2 |  | Y | 4 | ND | ND | ND | ND | ND | Y699_02127 |
| FAD/FMN-containing isoamyl alcohol oxidase MreA |  | Y | 5 | ND | ND | ND | ND | ND | Y699_07340 |
| Glutaminase GtaA |  | Y | 3 | ND | ND | ND | ND | ND | Y699_01844 |
| **Supplementary Table S3** (continued) | | | | | | | | | |
| Allergenic cerato-platanin |  | Y | 25 | ND | ND | ND | ND | ND | Y699_02605 |
| Cell wall protein |  | Y | 26 | ND | ND | ND | ND | ND | Y699_07973 |
| Hypothetical protein |  | Y | 27 | ND | ND | ND | ND | ND | Y699_00114 |

NS, no signal in q-PCR experiment; ND, not been detected; SP, signal peptide Y, yes, N, no; 2-DE, protein spots identified by two-dimensional gel electrophoresis and MALDI-TOF-MS/MS; In column Enzyme, x1-x15, xylanase indexes used in Figure 5.
